# Renal artery rupture following cutting balloon angioplasty for fibromuscular dysplasia: a case report

**DOI:** 10.4076/1757-1626-2-8881

**Published:** 2009-09-14

**Authors:** Elias N Brountzos, Nikolaos Ptohis, Helen Triantafyllidi, Irene Panagiotou, Themistoklis N Spyridopoulos, Evangelos P Misiakos, Alexios Kelekis

**Affiliations:** 1 Department of Radiology, Athens University Medical School, Attikon University Hospital, 1 Rimini St., 12462, Chaidari, Greece; 2 Department of Cardiology, Athens University Medical School, Attikon University Hospital, 1 Rimini St., 12462, Chaidari, Greece; 3 Department of Surgery, Athens University Medical School, Attikon University Hospital, 1 Rimini St., 12462, Chaidari, Greece

## Abstract

**Introduction:**

Angioplasty with the use of cutting balloons has been suggested by some case reports and small series for the treatment of renal artery stenoses that are resistant to conventional balloon catheters. Based on this limited experience, the use of this technology has been suggested as safe. Herein, we report a renal artery rupture following angioplasty with a cutting balloon. The complication was salvaged with a stent graft.

**Case presentation:**

A 30-year-old white female patient with resistant hypertension caused by a severe renal artery stenosis attributed to fibromuscular dysplasia, was submitted to conventional balloon angioplasty without success. Dilatation of the lesion with a cutting balloon resulted in arterial rupture, with concomitant retroperitoneal hematoma.

**Conclusion:**

Cutting balloon angioplasty of renal artery lesions resistant to conventional balloon angioplasty should not be considered as safe as previously thought. When proceeding with such a procedure, a stent graft should be available for immediate use.

## Introduction

Renal artery stenoses are the cause of hypertension in only 1% to 5% of the hypertensive patients. While atherosclerotic occlusive lesions consist the majority of renal artery lesions, the most common cause of renal artery lesions in young individuals is fibromuscular dysplasia (FMD). The most common type of FMD is medial fibroplasia, while intimal and perimedial FMD are less common [1,2].

Percutaneous balloon angioplasty is the first-line treatment for young individuals with hypertension caused by FMD renal artery stenoses [3]. Renal artery FMD lesions of the medial fibroplasia type respond favorably to balloon angioplasty with a technical success of 90% and clinical success rate of 85%, the other FMD types are more difficult to treat because they are more fibrotic and elastic [1]-[3]. Recently, few case reports and small series have introduced the cutting balloon angioplasty (CBA) in the management of non-atherosclerotic renal artery lesions that do not respond to conventional balloon angioplasty. These reports suggest that this technology is effective where the conventional approach has failed and is relatively safe [4]-[11].

We present a hypertensive female patient with a renal artery FMD lesion which was not responsive to conventional angioplasty and was treated with CBA. The lesion was dilated but the intervention was complicated by renal artery rupture and retroperitoneal hematoma. The patient was salvaged with the use of a stent-graft.

## Case presentation

Our patient is 30-year-old, white female, single, of Greek ethnicity. At the time of her admission her height was 170 cm, and weight was 62 kg. Her medical history and family history were unremarkable. She reported that she was not smoking or using alcohol.

**Figure 1:**
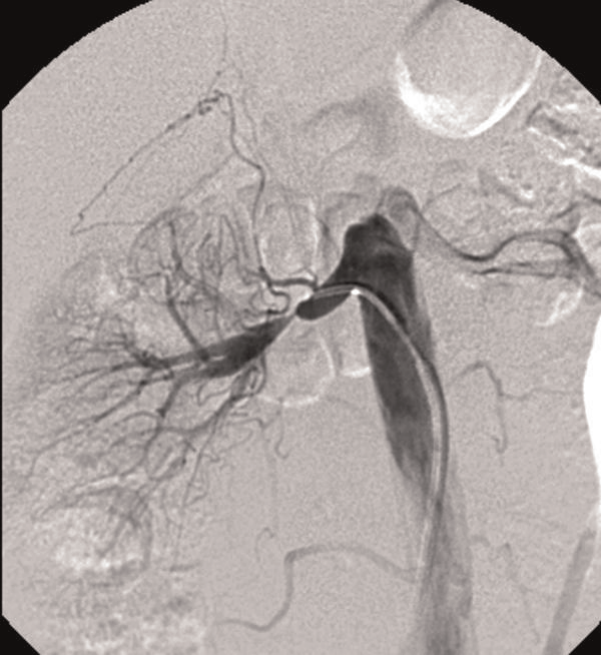
**Abdominal digital subtraction angiography of a 30-year-old female patient presenting with hypertension of recent onset depicts a high-grade stenosis at the trunk of the right renal artery**. Note that the lesion has characteristic features of the intimal type of fibromuscular dysplasia.

**Figure 2:**
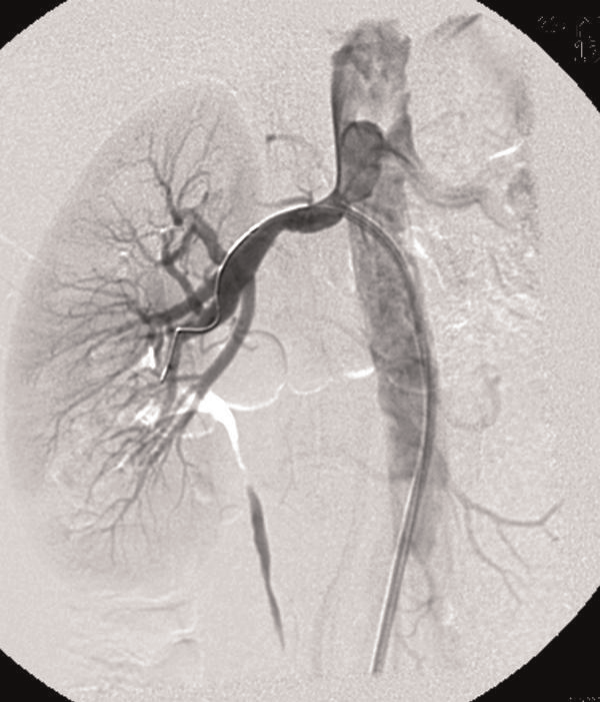
**Selective right renal artery angiography following dilatation of the lesion with a 5-mm and a 6 mm balloon depicts 50% residual stenosis**. The stenosis was accompanied with a 15 mm Hg of systolic pressure gradient.

One month prior to her admission in our institution she was diagnosed with hypertension (systolic 200 mmHg, diastolic 110 mmHg) at a recent routine medical examination. Renal magnetic resonance angiography depicted a significant truncal stenosis of the right renal artery. Medical investigation did not reveal any other cause of the hypertension. No medical therapy was started, instead she was referred to our interventional radiology service for evaluation and percutaneous treatment. After obtaining informed consent from the patient the procedure was initiated using local anesthesia. A 6-F sheath was introduced in the right common femoral artery and abdominal aortography and selective renal angiography were performed with standard diagnostic catheters and depicted a focal truncal lesion which caused high-grade stenosis (Figure 1). A 6-F guiding catheter (Veripath, Abbott Vascular, Diegem, Belgium) was then used to selectively catheterize the right renal artery ostium, and the lesion was crossed with a 0.018-inch platinum-tip guidewire. The lesion was dilated with a 5 × 20 mm balloon (Sterling™, Boston Scientific, Natick, MA, USA), at 14 atmospheres, but the waist could not be completely effaced. Repeat dilatation with a 6 × 20-mm balloon at 14 atmospheres also had inadequate effect. Control selective angiography depicted 50% residual stenosis, while pressure measurement after administration of 100 μg of nitroglycerine showed a 15-mmHg gradient of systolic blood pressure across the lesion (Figure 2).

The technical failure of the procedure predicted a clinical failure, so the decision to proceed with CBA was taken after discussing the risks and benefits with the patient. Also the vascular surgery department was informed and consented to this decision.

**Figure 3:**
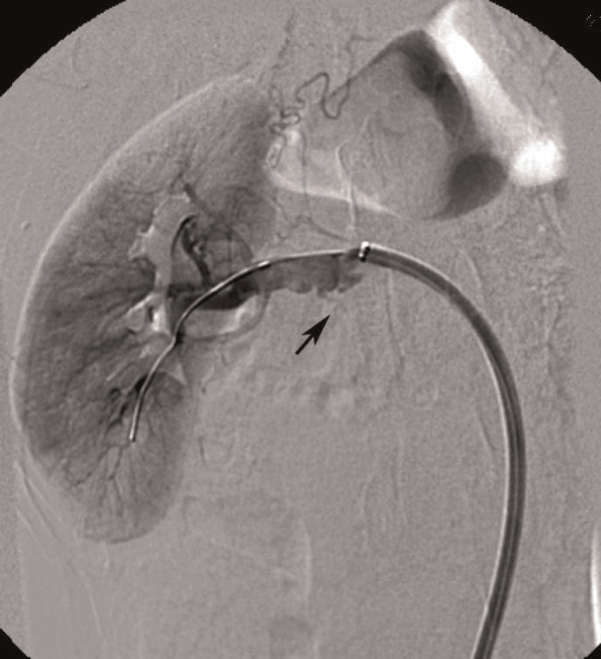
**Selective right renal artery angiography following dilatation of the residual lesion with a 6-mm cutting balloon depicts arterial dissection and contrast extravasation (arrow)**.

A 6 × 10 mm cutting balloon (Cutting balloon peripheral, Interventional Technologies Europe, Ltd, Ireland) was placed across the lesion and dilated at 6 atmospheres but the waist could not be effaced; then it was dilated at 8 atmospheres resulting into waist elimination. At this time point, the patient complaint of severe abdominal pain and she developed hypotension (60/40 mmHg). Intravenous fluids were instantly started with improvement of the hypotension. The balloon was removed, while control angiography depicted contrast extravasation from the renal artery; the stenosis was however eliminated (Figure 3). A 6-mm conventional balloon was reinserted and dilated for 5 minutes to tamponade the rupture. Again control angiography depicted continuation of the extravasation. The decision to place a stent-graft was made, while the vascular surgical team was notified and started their preparations.

**Figure 4:**
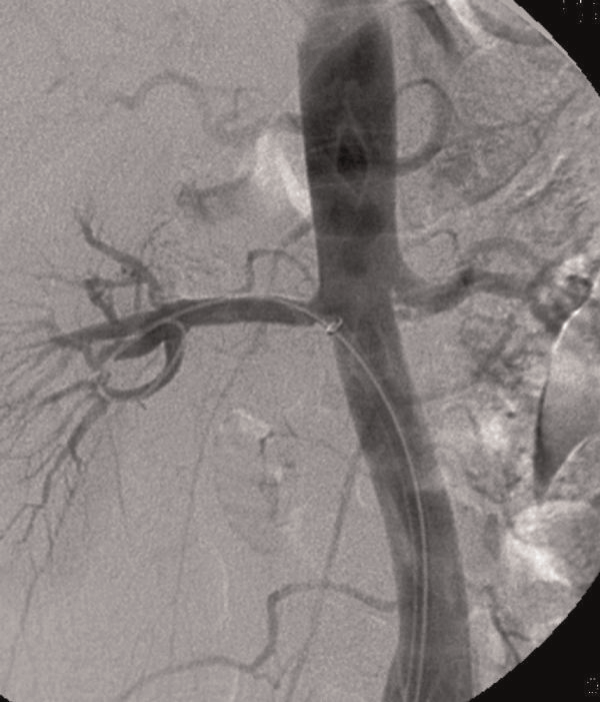
**Selective right renal angiography following the placement of a 6 × 25-mm self-expandable stent graft depicts elimination of the dissection and the extravasation, with restoration of the normal luminal patency**.

It was planned to select a stent-graft suitable for covering the rupture without compromising the branches of the renal artery. There was a 6 × 25 mm self expandable stent graft available in the department (Viabahn, W.L. Gore & Associates, Flagstaff, USA) which required a 9-F sheath for insertion, and could be inserted over a 0.035 inch guidewire. Following the required exchanges the stent-graft was successfully deployed from the ostium to the bifurcation of the right renal artery. Completion angiography showed elimination of the extravasation and of the renal artery stenosis (Figure 4).

**Figure 5:**
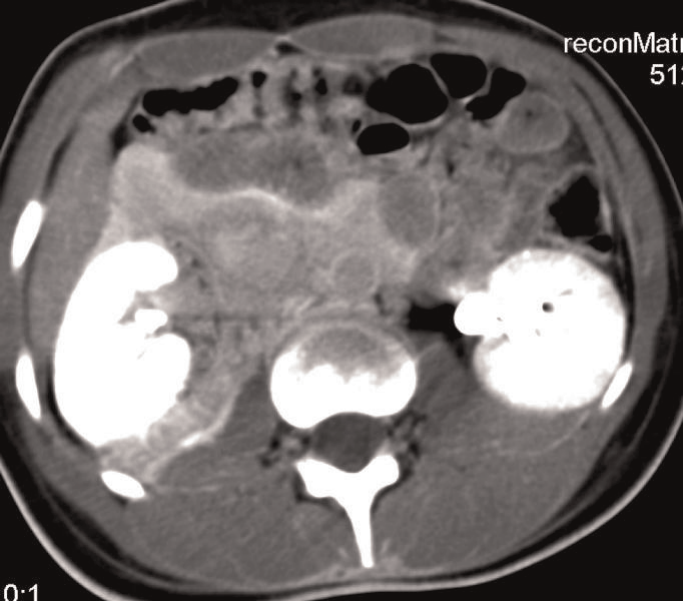
**Abdominal computer tomography following the procedure depicts a large peri- and pararenal hematoma**.

The patient was taken to the intensive care unit and she was well. Her hematocrite drop from 40% before the intervention, to 29% at the end of the procedure, but no blood transfusion was required. Abdominal computer tomography depicted a large peri-and pararenal hematoma (Figure 5). Her blood pressure normalized and she was discharged home after four days. She was instructed to receive acetylsalicylic acid 100 mg daily for life, and clopidogrel 75 mg daily for six months.

She undergoes regular clinical and ultrasonographic follow up. She is normotensive without medications while the Doppler ultrasonography of the right renal artery is normal one year after the intervention.

## Discussion

FMD is defined as an idiopathic non-atherosclerotic, non-inflammatory disease. Pathologically three types of FMD have been described: the medial fibroplasia which accounts for nearly 85% of the renal artery FMD lesions and is characterized by alternating webs and aneurysms; the intimal type which accounts for 5% and it is characterized by irregularly arranged mesenchymal cells within a loose matrix and fragmented elastic lamina; perimedial FMD consists approximately 10% of cases and is characterized by excessive tissue deposition at the junction of the media and adventitia [1,2].

Pathological angiographic correlation has shown that the alteration of web and aneurysms which produces the "string-of-beads" sign in angiography is typical of the medial type of FMD, while focal or tubular lesions cannot with certainty be attributed to specific histological types of FMD [2]. However, some authorities believe that a focal lesion is consistent with the intimal type of FMD [5].

Percutaneous balloon angioplasty is currently considered the first line treatment for young hypertensive patient with renal FMD lesions [2,3]. For the small subset of these patients, whose lesions are resistant to balloon angioplasty, there only therapeutic alternative was until recently open surgery.

Since its introduction in 1991, the indications of the CBA were the management of vascular stenoses that were resistant to conventional balloon angioplasty [12]. These among others include arterial stenoses in coronaries and pulmonary arteries, venous stenoses, and in-stent stenoses [13]. More recently, CBA has been used for peripheral arterial stenoses [14]. Haas et al first reported, in 2002, of a 12-year old boy with a stenotic lesion of the left renal artery that was successfully dilated with CBA. They reported of minimal extravasation that healed spontaneously [4]. To our knowledge, there are to date 15 patients reported for CBA treatment of renal artery lesions of various etiologies, while in seven of them the lesions were attributed to FMD with certainty [4]-[11]. In none of these seven patients the angiographic appearance was of the "beads-of-strings" type, the lesions were rather focal or tubular, suggestive of the hard-to-treat type of renal artery FMD [4,5,8]-[10]. In six of the seven patients CBA was successful, while in one there was a failure because the renal artery ruptured [8]. This patient was operated because balloon tamponade failed to seal the rupture, while attempts to use a stent graft failed because the authors used a balloon expandable device which was very rigid to be navigated into the tortuous renal artery. In three of the successful cases the procedure was complicated by an extravasation (n = 2) or a dissection (n = 1). In one patient who was followed up for a longer time the development of a false aneurysm was reported [9], while in the remaining no long term follow up is available.

Our patient suffered from hypertension caused by a hard-to-treat type of renal artery FMD, most probably the intimal type. Although histological confirmation was not available, on the basis of angiographic appearance our patient had similar lesion to that of Oguzurt et al. Our decision to proceed to CBA was encouraged by the increasing enthusiasm dwelling in the literature. Our fault might have been that we used a CBA with diameter matching the normal diameter of the renal artery, instead of using an undersized CBA to induce intimal incisions and then dilate further with a conventional balloon catheter [5]. We were able however to salvage the complication by using a self expandable stent-graft of the appropriate length and diameter.

## Conclusion

The take-home message from our case report is that the use of CBA for the management of resistant to conventional angioplasty renal artery FMD lesions should not be considered as safe as previously thought. Because salvage of a renal artery rupture with a stent graft is depended not only on their immediate availability in the interventional radiology suite, but also on the renal artery anatomy, we suggest that patients with FMD lesions resistant to conventional angioplasty should be given the option of open surgery, while cutting balloon angioplasty should be considered a second line treatment for this group of patients. Had the interventionist made the decision to proceed with CBA, he or she should use an undersized CBA, and leave a sheath in the renal artery ostium throughout the procedure, which could accommodate a stent-graft of the appropriate dimensions.

## Consent

Written informed consent was obtained from the patient for publication of this case report and accompanying images. A copy of the written consent is available for review by the Editor-in-Chief of this journal.

## Competing interests

The authors declare that they have no competing interests.

## Author's contributions

ENB was the first operator of the procedure, has analyzed the data and has written the manuscript; NP was the second operator, and has performed literature search; HT and IP have evaluated the patient clinically initially and during follow and have contributed to the writing of the manuscript; TNS has critically analyzed the literature data; EPM, and AK have critically evaluated the data and edited the manuscript. All authors have read and approved the final manuscript.
